# Female- or male-biased offspring generated by targeted distortion of sex chromosome transmission in the mouse

**DOI:** 10.1093/genetics/iyaf246

**Published:** 2025-11-20

**Authors:** Hermann Bauer, Frederic Koch, Bettina Lipkowitz, Jürgen Willert, Gaby Bläß, Manuela Scholze-Wittler, Sandra Währisch, Lars Wittler, Bernhard G Herrmann

**Affiliations:** Max Planck Institute for Molecular Genetics, Ihnestr. 63-73, Berlin 14195, Germany; Max Planck Institute for Molecular Genetics, Ihnestr. 63-73, Berlin 14195, Germany; Max Planck Institute for Molecular Genetics, Ihnestr. 63-73, Berlin 14195, Germany; Max Planck Institute for Molecular Genetics, Ihnestr. 63-73, Berlin 14195, Germany; Max Planck Institute for Molecular Genetics, Ihnestr. 63-73, Berlin 14195, Germany; Max Planck Institute for Molecular Genetics, Ihnestr. 63-73, Berlin 14195, Germany; Max Planck Institute for Molecular Genetics, Ihnestr. 63-73, Berlin 14195, Germany; Max Planck Institute for Molecular Genetics, Ihnestr. 63-73, Berlin 14195, Germany; Max Planck Institute for Molecular Genetics, Ihnestr. 63-73, Berlin 14195, Germany

**Keywords:** genetically modified organism, sex-ratio, transmission ratio distortion, *t*-haplotype, sex chromosome, selfish gene

## Abstract

In animal breeding, female or male progeny is preferred depending on the use of the animals. Especially in livestock, offspring with undesired sex is of low economic value and often culled. Here we achieve targeted sex ratio distortion in the mouse, leveraging components of a selfish supergene, the *t*-haplotype. Utilizing its central element, the *t*-*complex-responder*, we generate a highly “selfless” genetic element disabling transgenic sperm. When integrated on the X or Y chromosome, it results in a high prevalence of nontransgenic offspring of the desired sex. Our strategy overcomes key limitations of existing strategies, including compromised animal health, reduced fertility, and fully transgenic breeding stocks, which might foster acceptance of its application to livestock. The efficacy of our approach marks a groundbreaking advance in animal breeding.

## Teaser

The mouse t-haplotype is a selfish genetic element strongly promoting its transmission from t/+ males to their offspring. Since in animal breeding, females or males are usually preferred depending on their use, Bauer et al. figured that the central component of t, *SmokTcr*, could be used for biasing the inheritance of sex chromosomes. *SmokTcr* alone reduces the inheritance of the chromosome carrying it by disabling the motility of the respective sperm. Therefore, they integrated *SmokTcr*- constructs on either the X or the Y chromosome of male mice, and thereby achieved a high excess of non-transgenic male or female offspring. This approach might find application to livestock.

## Introduction

Animal production faces major challenges due to limited resources for a growing human population and increasing standards for animal welfare. A substantial improvement in breeding efficiency and mitigation of ethical problems could be achieved by preferential inheritance of desired genetic traits. This is particularly relevant in livestock breeding, where almost half of the progeny with undesired sex often has little or no economic value and is culled, leading to both economic and ethical issues (Douglas and Turner 2020).

Controlling the sex of progeny, therefore, is of paramount interest. Several approaches for altering the sex ratio have been developed, with pioneering work conducted in the mouse model. These include transgenic sex reversal by *Sry* knock-out or sex chromosome shredding, which, however, often resulted in animals with reproductive defects (Zuo et al. 2017; Kurtz et al. 2021) and failed to yield sex ratio distortion (Bunting et al. 2024).

Gene drives, while theoretically applicable for inducing transgenic sex reversal, have shown low efficiency in mammals, highlighting the challenges of adapting strategies successfully used for insects to mammals (Grunwald et al. 2019; Weitzel et al. 2021). In a recent approach in the mouse, selective lethality of embryos carrying the Y chromosome is induced by the combined inheritance of an autosomal Cas9 transgene and a Y-chromosomal guide-RNA cassette directed against essential embryonic genes (Yosef et al. 2019). Due to incomplete penetrance, some animals develop to term and are malformed, raising animal welfare concerns. Another, more recently developed synthetic lethal system induces embryonic death of all progeny with undesired sex prior to implantation (Douglas et al. 2021). However, all parents and offspring are transgenic and the productivity of the breeding stock is strongly reduced.

Biasing offspring sex based on X/Y sperm manipulation can avoid the drawbacks of the approaches mentioned above. Incubation of sperm with antibodies against a Y-sperm specific epitope, sperm pretreatment with undisclosed substances (reviewed in Quelhas et al. (2023)), or manipulation of Toll-like receptors *in vitro* (Umehara et al. 2019; Ren et al. 2021) requires extensive manipulation of sperm samples and, to some degree, are controversial with respect to their effectiveness. In cattle, fluorescence-activated cell sorting (FACS) is used to separate X- and Y-chromosome-bearing sperm for artificial insemination (Holden and Butler 2018; Obuchi et al. 2019). However, sperm sorting technology is costly for the breeder and not well established in other livestock species. A genetic system designed toward generating a surplus of nontransgenic progeny has been presented recently in a preprint (Yosef et al. 2025). It employs dCas9-mediated suppression of a gene required for spermatid maturation in the mouse, impairing the fertilization capability of transgenic sperm. Transgenic males express dCAS9-Krab as well as all guide RNAs ubiquitously in transgenic males and are growth impaired, though their sperm effectively causes sex ratio distortion (SRD) in favor of female offspring (Yosef et al. 2025).

Several naturally occurring genetic systems influencing transmission ratio in their favor have been reported (Burt and Trivers 2006), some of which hold promise for a practical application. The most prominent element causing transmission ratio distortion in mammals is the mouse *t*-haplotype, a naturally occurring variant of chromosome 17, able to strongly increase its transmission from *t/+* heterozygous males to their offspring at the expense of the wild-type chromosome (Lyon 2003). The *t*-haplotype achieves high transmission by a “poison-antidote” mechanism. Its “poison” genes, so-called *t*-distorters, act harmfully by affecting progressive movement in all sperm (Bauer et al. 2005, 2007, 2012; Amaral and Herrmann 2021). The “antidote”, termed *t*-complex-responder (*Tcr*), encodes a dominant-negative form of *Smok* (*Smok^Tcr^*), able to rescue progressive motility. But it does so only in sperm carrying the gene providing them with a fertilization advantage over their competitors (Herrmann et al. 1999).

However, in the absence of *t*-distorters, the dominant negative action of *Smok^Tcr^* results in a disadvantage for sperm. This low transmission ratio can be phenocopied by *Tcr* transgenes. We have been the first to report successful SRD in a mammal (Herrmann et al. 1999). Using a *Tcr* transgene randomly integrated on the Y chromosome, we achieved a surplus of male offspring at a 2:1 (male/female) ratio, but only in combination with *t*-distorters. In contrast, in the present study, we used single-copy integrations of *Tcr* transgenes into defined landing sites on the X or Y chromosome. This allowed manipulation of the sex ratio by design. We optimized the *Tcr* transgene to further increase its efficiency from 65% to up to 88% nontransgenic offspring of the desired sex, again without using distorters. This is the first report demonstrating highly efficient sex ratio distortion in mice in favor of either male or female nontransgenic progeny.

## Materials and methods

### RNA-Seq

For RNA-isolation, a testis piece, freshly isolated or stored at −80 °C was homogenized using a tissueLyser (Qiagen cat. 85220) in a 2 ml Eppendorf tube, 1 min, frequency 30.0.

Total RNA prepared with Trizol (Invitrogen, Thermo Scientific, see below) was DNAse digested and further purified using the RNeasy Micro kit (Qiagen). Any residual genomic DNA was digested on column according to manufacturer's instructions, with the addition of an extra 1 µl of RNase-free DNase I (Roche). The RNA was eluted using RNase-free water, quantified using the Qubit RNA HS Assay (Life Technologies), and the integrity was verified using Bioanalyzer RNA Pico chips (Agilent).

Approximately 150–200 ng of total RNA was used for the generation of strand-specific RNA-seq libraries using the ScriptSeq v2 (Epicentre) low-input library preparation kit according to manufacturer's instructions. The RNA-seq libraries were quantified using the Qubit high-sensitivity DNA assay (Life Technologies) and the size distribution was verified using the DNA HS Bioanalyzer chips (Agilent). Libraries were paired-end sequenced on a HiSeq 2000 (Illumina) with 2 × 50 bp read length.

### ChIP-seq

We isolated single cells from mouse testes according to Getun et al. (2011). Crosslinking of approximately 1 × 10^6^ cells was performed in PBS essentially as previously described (Koch et al. 2017). Cells were washed twice with cold PBS containing 0.05% Triton X-100, pelleted, snap frozen, and stored at −80 °C until sonication.

Cells were processed using the iDeal ChIP-Seq kit (Diagenode) according to manufacturer's instructions. Sonification was performed on a Bioruptor Pico (Diagenode) using 3 runs of 10 cycles (30 s on, 30 s off) in a 4 °C water bath. Sheared chromatin was purified and the size distribution was verified using a DNA HS Bioanalyzer chip (Agilent). Approximately 200,000 cells were used for ChIP with the anti-H3K27Ac (ab4729, Abcam) antibody.

Approximately 1–5 ng of ChIP DNA was used to generate libraries using the TrueSeq ChIP-Seq kit (Illumina) with minor modifications. After adapter ligation, a 0.95× volume of AMPure XP beads (Beckman Coulter) was used for a single round of purification. After the addition of 1 µl primer mix (25 mM each, Primer 1: 5′-AATGATACGGCGACCACCGAG-3′; Primer2: 5′- CAAGCAGAAGACGGCATACGAG-3′) and 15 µl 2× Kapa HiFi HotStart Ready Mix (Kapa Biosystems), amplification was performed for 45 s at 98 °C, 5 cycles of [15 s at 98 °C, 30 s at 63 °C and 30 s at 72 °C] and a final 1 min incubation at 72 °C. The PCR products were purified using a 0.95× volume of AMPure XP beads. The libraries were amplified for an additional 13 cycles, purified using a 0.95× volume of AMPure XP beads, quantified using the Qubit DNA HS assay, and the size was validated using DNA HS Bioanalyzer chips (Agilent). Libraries were sequenced on a NextSeq500 (Illumina) with 1 × 75 bp read length.

### Genome assemblies

Datasets were mapped to the Mus musculus GRCm38/mm10 genome assembly containing chromosomes 1–19, X, Y, and M and the refSeq annotations (UCSC) in refflat gtf format.

### Bioinformatic analysis

RNA-seq reads were mapped with TopHat2 (version 2.1.0) (Kim et al. 2013) using bowtie (version 1.1.2) (Langmead et al. 2009), providing refSeq annotations and the options “–no-coverage-search –no-mixed –no-discordant -g1 –library-type fr-secondstrand”. For visualization, wiggle tracks were generated with BEDTools (version 2.23.0) (Quinlan and Hall 2010), converted into bigwig format, and loaded into the Integrated Genome Browser (Freese et al. 2016). FPKM's were calculated using Cuffdiff, part of Cufflinks (version 2.2.1) (Trapnell et al. 2010, 2012), with the options “-u –no-effective-length-correction -b”.

ChIP-seq data were mapped using bowtie (version 1.1.2) with the options “-m 1 -S -y”. We then used MACS (Zhang et al. 2008) to determine the average fragment length of the sequenced samples and a custom Perl script to elongate the mapped reads to this length. Duplicates were then removed and .wig files were generated using BEDtools (version 2.23.0) (Quinlan and Hall 2010). The files were converted into bigwig format and loaded into the Integrated Genome Browser (Freese et al. 2016).

### Identification of spermiogenesis-specific promoters on X

Genes with a haploid stage-specific expression pattern were selected based on the generated FPKM (Fragments Per Kilobase per Million mapped fragments) values of the staged testis RNA-Seq data. We then isolated genes specific for d16 after birth (p.p.) with an FPKM value of <2 at d12 and ≥ 2 at d16, as well as those for d24 with an FPKM value of <2 at d12 and d16 and ≥2 at day 24. We set a cutoff at expression value FPKM < 30.

For the analysis of tissue-specific expression, we obtained CAGE-Seq data from 35 adult mouse tissues (accession E-MTAB-3579; https://www.ebi.ac.uk/biostudies/arrayexpress/studies/E-MTAB-3579) and selected genes with detectable expression in either testis and/or epididymis and removed those with a combined TPM (tags per million) score across all other 33 tissues of more than 1.

To obtain the final list of genomic regions, we then selected the overlap between haploid stage-specific and tissue-specific genes, extracted their respective promoters (−2 kb to TSS), and removed those promoters of genes located on autosomes.

### Transgenes

To obtain TgY2, we cloned the promoter fragment of *Cypt1* upstream of the 5′-utr and coding sequence of *Tcr^t6^* (Herrmann et al. 1999). We attached the 3′-utr of *Smok^Tcr^* and a SV40 poly-A signal and flanked the construct with homology regions (HRs) for integration in the vicinity of the *Zfy2* gene. TgY3 is identical to TgY2 but carries in addition a putative enhancer sequence identified by ChIP-seq analyses (see above). We PCR-amplified the putative enhancer sequence by PCR and inserted it 5′-upstream of the promoter. To derive TgY4, we sequence-optimized the open reading frame of TgY3. For TgX2, we replaced the *Zfy2* HRs as well as the guide-RNA target sequences in TgY4 for sequences close to *Akap4* and omitted the putative *Cypt1* enhancer present in TgY3 and TgY4. Cloning primers are in Supplementary Table 1.

### ES cell culture, genetic engineering, and analysis

We carried out embryonic stem cell (ESC) culture of G4-F1 hybrid ES cells (gift of A. Nagy) (George et al. 2007) on mitotically inactivated embryonic fibroblasts according to standard procedures (Ramirez-Solis et al. 1993).

We integrated transgenic constructs via homologous recombination, stimulated by CRISPR/Cas-mediated DNA cleavage. Guide RNA sequences were designed using CRISPOR (http://crispor.tefor.net/) (Concordet and Haeussler 2018). Oligonucleotides were annealed and ligated into the pX330 vector digested with BpiI (pX330-U6-Chimeric_BB-CBh-hSpCas9 plasmid (Cong et al. 2013), gift from Feng Zhang, Addgene plasmid #42230; http://n2t.net/addgene:42230; RRID:Addgene 42230).

We confirmed correct, singlecopy integration of the transgenes by Southern blotting of ES-cell clones using external genomic- or transgene-specific probes, respectively. Alternatively, we verified single-copy integration in breedings. After expansion of Southern-blot positive clones, we performed full-length PCR amplification of the integrated transgene using Prime STAR GXL DNA polymerase (TAKARA) with primers outside the integration site and sequenced the PCR product of the integrated transgene.

### Generation, husbandry, and transmission test of mouse lines

We expanded correctly targeted ESC clones from frozen 96-well plates to 3.5 cm dishes, froze stocks, and, after confirming the sequence of the transgene insertion by PCR (see above), used the clone for ESC aggregation with diploid morulae in the transgenic facility of the MPIMG (Artus and Hadjantonakis 2011). Mouse lines were established by backcrossing to wild-type strains such as C57BL/6J.

All animal procedures were in accordance with institutional, state, and government regulations (LAGeSo Berlin, animal licenses G0243/18 and G0098/23 for aggregation experiments and G0309/18 and G0186/23 for G0 animals and the resulting mouse lines).

To determine sex ratio and transgene transmission ratio from the transgenic lines, transgenic males were mated with wild-type females. In the mornings of the following days, females were checked for copulatory plugs and, if plug-positive and pregnant, were sacrificed at 13.5 d after conception. Biopsies of embryos were lysed in Laird's buffer (Laird et al. 1991) and genotyped by PCR using transgene-specific primers and sexing primers (Clapcote and Roder 2005) (Supplementary Table 1).

We performed statistical tests of the observed transgene transmission rate in offspring, relative to the expected Mendelian transmission rate of 50%.

### Transgene expression test

Before establishing and breeding the line, a G0 chimeric male was used for the transgene expression test. We euthanized sexually mature males by CO_2_ asphyxiation (GasDocUnit, Medres) and isolated testes for RNA isolation and *in situ* hybridization on paraffin sections using the RNAscope 2.0 HD Detection Kit (Brown), (ACD, Bio-Techne) as suggested by the manufacturer, except for a longer incubation step in Amp5 solution (60 min instead of 30 min). Staining times were up to 2 h and adjusted according to signal strength.

We isolated total RNA prepared from testis tissue samples using Trizol (Invitrogen, Thermo Scientific). We removed genomic DNA contamination with the DNA-free kit AM 1906 (Ambion, Invitrogen, Thermo Fisher Scientific). RNA was quantified on a NanoDrop device (NanoPhotometer, IMPLEN, Version 1.0). We analyzed RNA quality by gel electrophoresis or with Bioanalyzer 2100 (Agilent).

We performed cDNA synthesis for RT-qPCR analysis using 1 μg of testis RNA with the SuperScript reverse transcription system (Invitrogen) or M-MLV Reverse Transcriptase (Promega).

Primers were designed with Primer3plus. To generate specific, discriminating primers, we used PrimerBLAST and carried out sequence alignment with SnapGene. We used Gapdh as a reference gene (Gong et al. 2014). Primers are listed in Supplementary Table 1.

DNA fragments were used directly or, if required, subcloned by ligation of blunt-end PCR fragments into pBSSK linearized with EcoRV. After blue-white selection of colonies, we analyzed plasmids by restriction digest and Sanger sequencing.

### Statistical analysis

For expression tests of (trans)genes by RT-qPCR, bars represent the average relative expression value, error bars represent the standard error of the mean of 3 technical replicates.

For the transmission test, we performed two-sided binomial tests against the expected Mendelian rate of 50%. Absolute offspring numbers (*n*) are given in graphs and tables.

For comparison of TgY3 vs TgY2 values, we applied a Fisher's exact test.

## Results

### Biasing sex ratio and maintaining health and fertility

To achieve targeted sex-ratio distortion without affecting the health or fertility of the animals, several conditions have to be met. Transgene expression should be restricted to transgenic sperm and therefore occur post-meiotically in haploid spermatids only. To achieve that, the promoter driving transgene expression should be spermatid-specific, and transgene-derived mRNA and proteins should be retained in the transgenic spermatids, a challenging requirement given that spermatids typically share mRNA due to their development in a syncytium (Braun et al. 1989). Notably, *Smok^Tcr^* is the only case where nonsharing has been demonstrated experimentally (Veron et al. 2009). In addition, transgene activity in somatic cells which might compromise the health of the animal, should be avoided. Moreover, the expression level of the transgene on a sex chromosome must be high enough to achieve an effect. Since the X and the Y chromosome undergo meiotic sex chromosome inactivation (MSCI), resulting in silencing of many regions (Turner 2007), transgene expression levels may be affected. However, excessive expression might allow sharing of Tg-derived mRNA between spermatids to some degree, which might reduce the TRD effect. Furthermore, the transgenic construct should not interfere directly or via chromatin silencing with the expression of an endogenous gene, which might compromise the health or fertility of the transgenic male. Hence, integration into transcription units should be avoided.

To achieve targeted inheritance of desired genetic traits, we first established site-directed, single-copy and selection-marker-free integration of *Smok^Tcr^* constructs. Such transgenes, when integrated in the vicinity of the *Smok2b* locus, showed TRD to a similar degree as randomly integrated transgenes (data not shown). To achieve sex ratio distortion, we integrated *Smok^Tcr^* transgenes controlled by autosomal promoters on the X and Y chromosomes. However, these transgenes showed very low expression and no sex ratio distortion (data not shown). We reasoned that this might be due to silencing of transgene promoters by chromatin adjacent to the transgene integration sites.

### Finding landing sites escaping MSCI on X and Y chromosomes

**Fig. 1.**
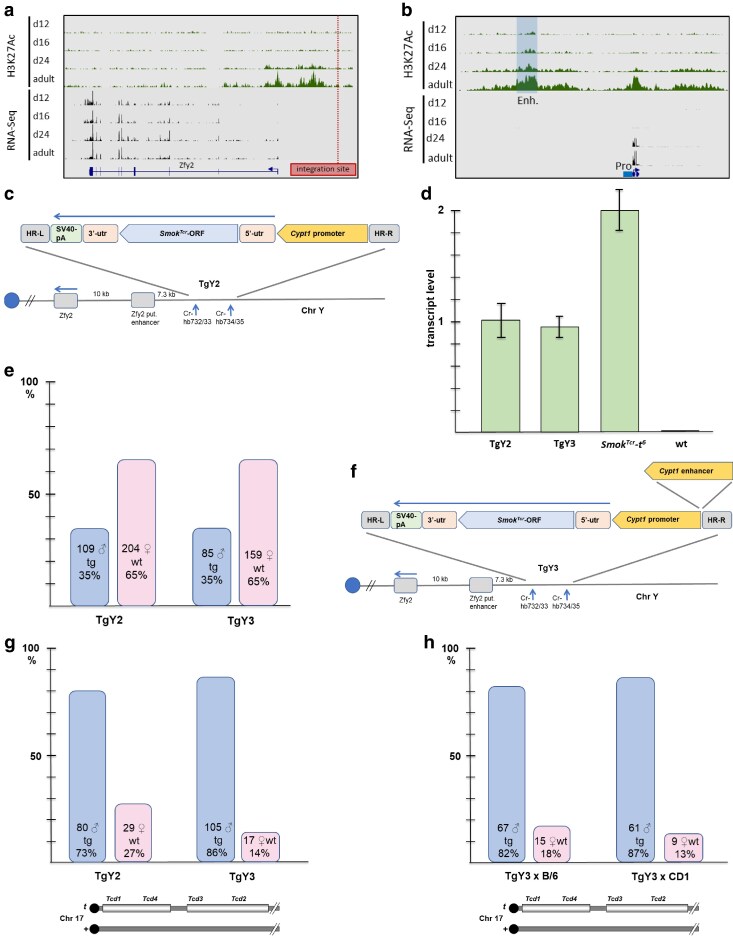
Targeted sex ratio distortion by a transgene integrated on the Y chromosome. a) Identification of a suitable integration site near the *Zfy2* gene based on histone modification data. The H3K27Ac signatures and expression profiles in post partum testes indicate gene expression from an upstream exon from d24 on and identify nearby active chromatin regions and a landing site for our transgenes. b) ChIP-seq and RNA-seq identify the *Cypt1* promoter and a putative *Cypt1* enhancer for transgene expression. c) TgY2, a *Smok^Tcr^* transgene integrated into the landing site near *Zfy2* identified in (A). d) Analysis of Tg expression by qPCR, in comparison to endogenous *Smok^Tcr^*. e) Tg transmission and sex ratio in offspring from males carrying the indicated Tg construct. f) TgY3, a *Smok^Tcr^* transgene carrying in addition a putative *Cypt1* enhancer integrated into the landing site near *Zfy2*. g) Analysis of Tg transmission and sex ratio in offspring from males carrying *t*-distorters encoded on the *t*-haplotype *t^h51^*-*t^h18^* on chromosome 17 in addition to the indicated Tg construct on the Y chromosome. h) TgY3 transmission and sex ratio after backcrossing to different genetic backgrounds (B/6 or CD1).

Finding an appropriate landing site outside gene bodies and active during spermiogenesis is challenging. We looked for landing sites in regions that become active during spermiogenesis, based on epigenetic chromatin signatures. To pinpoint such regions, we performed Chromatin Immunoprecipitation Sequencing (ChIP-seq) experiments on nuclear material isolated from mouse testis at 3 time points after birth (*post partum*; p.p.): day (d) 12, 16, 24, and from adult testis. The first wave of spermatogenesis starts right after birth, and therefore, this first differentiation process can be staged. Day 12 marks the meiotic stage, day 16 the onset of spermiogenesis (the haploid stage), and day 24 a later stage of spermiogenesis in which a particular set of genes, including members of the *Smok* gene family, are first expressed. Additionally, we performed RNA-sequencing (RNA-seq) of staged testes (Table 1). The combination of ChIP-seq and RNA-seq data allowed us to identify suitable landing sites. We chose a region upstream of *Zfy2* on the Y chromosome (LS-*Zfy2*), which becomes active as indicated by H3K27 acetylation (upregulated between d24 and adult; Fig. 1a; Table 1).

**Table 1.** Temporal expression profile of post-meiotically activated genes determined by RNA-seq of staged testis samples.

Gene	day 12 p.p.	day 16 p.p.	day 24 p.p.	adult
*Smok2a*	0	0	1.12	6.46
*Smok2b*	0	0	1.05	5.59
*Cypt1*	0	0.36	14.12	55.72
*Akap4*	0.28	0.11	98.61	564.58
*Zfy2*	5.44	5.45	4.85	9.84

Numbers are FPKM values.

### A promoter/enhancer for post-meiotic transgene expression

We hypothesized that a promoter escaping X/Y inactivation and becoming active on the X or Y chromosome in late spermiogenesis should be better suited for achieving stage-specific transgene expression at a sufficiently high level than the autosomal *Smok^Tcr^* promoter.

To identify such a promoter, we searched for genes expressed at moderate levels starting from d24 p.p. and exceeding *Smok2a*/*Smok2b* expression levels. Since most Y-expressed genes activated during spermiogenesis are repetitive and promoter strength is therefore difficult to assign to a particular gene locus, we focused on single-copy genes located on the X chromosome.

We found that the *Cypt1* promoter fulfilled all criteria discussed above (Kitamura et al. 2004). It is spermiogenesis-specific and expressed from day 24 p.p. at a moderate level (Fig. 1b, Table 1). This promoter was used to drive the expression of a *Smok^Tcr^* coding sequence flanked by a 5′- and 3′-UTR and the SV40 poly(A) signal (TgY2; Fig. 1c). Using CRISPR/Cas9, we integrated the transgene into LS-*Zfy2* in mouse ES cells, generated mice via morula aggregation, and evaluated stage- and site-specific transgene expression via RT-qPCR of testis RNA.

The transgene displayed restricted spermatid-specific expression, albeit at approximately half the expression level of the *t*-haplotype *Smok^Tcr^*, though the expression level of *Cypt1* is about 10-fold the level of *Smok2b*, the wild-type ortholog of *Smok^Tcr^* (Fig. 1d, Table 1). This may be due to unfavorable post-transcriptional regulation or could indicate some degree of attenuation of the promoter, possibly due to a lack of regulatory elements required for full activation in its ectopic environment.

Nonetheless, we observed significant sex ratio distortion favoring nontransgenic females (65%; Fig. 1e). We aimed to increase expression of the transgene by adding an enhancer sequence. Using our ChIP-seq data, we identified a putative enhancer region located upstream of *Cypt1*, which becomes active when the gene is first transcribed, as based on histone modification data (H3K27Ac+), indicating active enhancers (Fig. 1b). We integrated the putative enhancer sequence into TgY2 upstream of the promoter driving *Smok^Tcr^* expression and generated the mouse line TgY3 (Fig. 1f). TgY3 males showed the same expression level as TgY2 males and generated 65% wild-type female offspring, just like TgY2 (Fig. 1, d and e). Therefore, the enhancer had no effect on the expression level of the transgene.

We then crossed both lines to a sensitized genetic background containing a partial *t*-haplotype, *t^h51^*-*t^h18^* expressing *t*-distorter genes causing high transmission of *Smok^Tcr^* alleles (Lyon 1984). TgY3 showed significantly higher transmission than TgY2 (86% vs 73% males, Fisher's exact test statistic value 0.0205, Fig. 1g, Table 2), indicating a marked effect of the enhancer, though the underlying mechanism is not clear.

**Table 2.** Tg-transmission and sex-ratio distortion.

Line	tg	Non-tg	total	%tg	%non-tg females	Binomial test
TgY2; +/+	109	204	313	35	65	8.65E−08
TgY2/0; *t^h51^*/*t^h18^* N1	80	29	109	73	27	1.09E−06
TgY3/0; +/+ G0	85	159	244	35	65	2.50E−06
TgY3/0; *t^h51^*/*t^h18^* N1	105	17	122	86	14	1.15E−16
TgY3/0; *t^h51^*/*t^h18^* N2, N3, N4 × B/6	67	15	82	82	18	5.26E−09
TgY3/0; *t^h51^*/*t^h18^* N1, N2, N3 × CD1	61	9	70	87	13	1.28E−10
TgY4/0; G0	25	181	206	12	88	2.24E−30

Line	tg	Non-tg	total	%tg	% non-tg males	Binomial test
TgX2/0; G0	53	188	241	22	78	6.67E−19
Controls	males	females	total		% males	
Offspring from control G0 males	278	267	545		51	0.67
Offspring from control N1, N2, N3 × B/6 males	214	201	415		52	0.56

Transmission rates of *Smok^Tcr^* -transgenes (tg/0; +/+) without or in presence of distorter factors (tg/0; *t^h51^*/*t^h18^*).

We performed a two-tailed binomial test, comparing to expected Mendelian chromosome transmission (50%). Abbr.: G0 stands for founder generation, N for backcross generation.

TRD of *t*-haplotypes has been shown to be highly variable depending on the genetic background (Gummere et al. 1986). Therefore, we backcrossed TgY3 together with *t^h51^*–*t^h18^* to the inbred strain C57BL/6J (B/6) and to an outbred strain with high fertility, CD1. Transmission tests yielded 82% and 87% male offspring, respectively, indicating similarly high effects of both transgenes on either background.

The combined data showed that both transgene constructs are effectively causing SRD, either in favor or at the disadvantage of the Tg-carrying chromosome. The effect is stable through generations and preserved on different genetic backgrounds (Table 2). The rescue effect of *Smok^Tcr^* in the presence of *t*-distorters generated more pronounced deviations from the Mendelian sex ratio than *Smok^Tcr^* transgenes in a wild-type background.

### Optimizing *Smok^Tcr^* improves the sex-bias in progeny

**Fig. 2.**
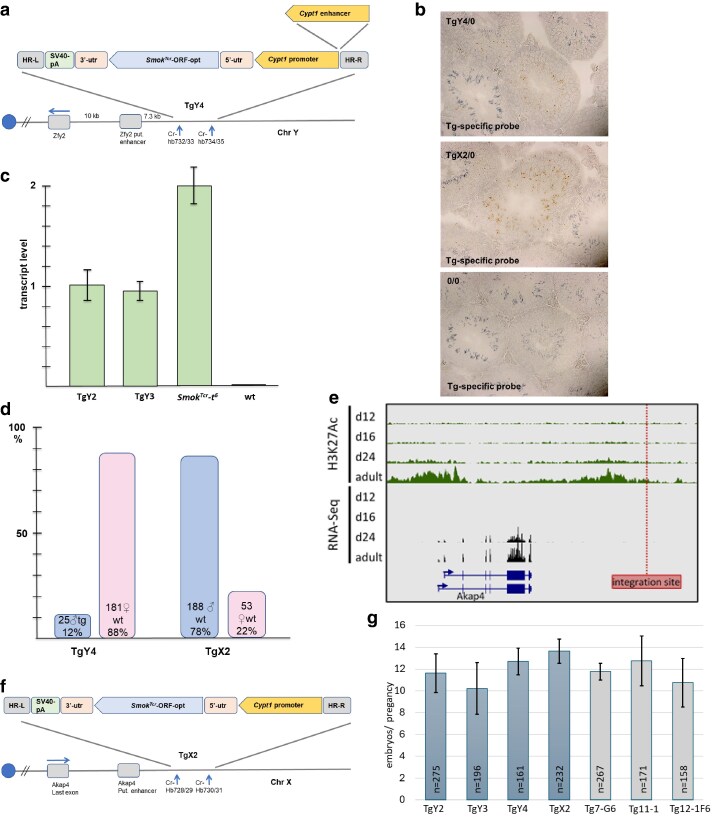
Enhanced sex-ratio distortion by an improved transgene integrated on the Y or the X chromosome. a) Structure of the Y-chromosomal *Smok^Tcr^* -transgene TgY4. The *Cypt1* promoter and enhancer were utilized to drive the expression of an improved Tg cassette integrated in LS-*Zfy2*. b) TgY4 or TgX2 expression detected by *in situ* hybridization of testis sections is observed in spermatids. c) TgY4 expression detected by RT-qPCR of testis RNA, in comparison to endogenous *Smok^Tcr^*. d) Tg inheritance and sex in offspring from TgY4 or TgX2 males indicate highly efficient sex ratio distortion. e) Identification of a transgene integration site in the vicinity of the *Akap4* locus (LS-*Akap4*). f) Structure of the transgene TgX2 integrated near *Akap4*. g) Fertility of transgenic lines showing sex ratio distortion (dark grey) and control lines (light grey). Bars show average numbers of embryos per pregnancy, error bars show standard deviation between males. *n* = total embryos scored. Numbers are from Table 3.

The most wanted practical application of SRD is low transmission of a transgene integrated on the unwanted sex chromosome, resulting in high transmission of the preferred, nontransgenic sex chromosome. However, as shown above, this “low-ratio” phenotype is harder to obtain than high transmission. Therefore, we explored whether transgene transmission could be further lowered by increasing *Smok^Tcr^* activity. To this end, we codon-optimized TgY3 to obtain TgY4 (Fig. 2a). We generated a transgenic line and tested for expression by *in situ* hybridization on testis sections, confirming appropriate site- and stage-specific expression, and by RT-qPCR (Fig. 2, b and c). The breeding test showed 88% female progeny, an unprecedented level of sex-ratio distortion with *Smok^Tcr^* and a 3.9-fold improvement of the female: male ratio (1.85× vs 7.33×) over TgY3 (Fig. 2d, Table 2).

For certain applications, a surplus of male offspring is desirable. Therefore, to adapt this approach accordingly, we developed an X-integrated, codon-optimized *Smok^Tcr^* transgene, TgX2. Leveraging our RNA-seq and ChIP-seq datasets, we identified a suitable transgene landing site on the X chromosome downstream of *Akap4* (LS-*Akap4*), a region activated late during spermiogenesis (Fig. 2e, Table 1). As a putative enhancer region marked by increasing levels of H3K27Ac during spermiogenesis is present near the integration site, we did not include any additional enhancer sequence in our transgene (Fig. 2f).

After generating the transgenic line TgX2, we confirmed appropriate transgene expression (Fig. 2c). Testing for sex ratio distortion demonstrated that TgX2 produced 78% male, nontransgenic offspring (Fig. 2d, Table 2).

### Transgenic males are healthy and fertile

Besides a strong and stable SRD effect, the value of a transgenic line highly depends on the health and fertility of the transgenic males.

A transgene might affect the well-being of a carrier animal by unintended consequences of integration, such as on- or off-target effects during CRISPR/Cas-mediated integration or, less likely, genomic lesions caused by integration. In addition, transgene expression can be detrimental to the animal's health. To address issues of general well-being, we scored G0 founder animals as well as N1 and N2 backcross animals according to the guidelines of the German Federal Institute for Risk Assessment (BfR). None of our transgenic lines showed a correlation between transgenic status and health issues of animals. Thus, neither our integration procedure nor the transgenes had a negative impact that manifests in this scoring.

For the applicability of our approach in animal breeding, the fertility parameters of transgenic males are of particular interest. Generally, matings of laboratory mouse strains show a significant variability in plug rates, proportion of successful plugs (total plugs/pregnancies), and number of embryos and offspring. Not only the performance of a specific breeder male, but also other factors are important, such as the age and physical status of the female and conditions in the animal facility. We compared the average number of embryos produced in test matings by transgenic males from our SRD lines with those from transgenic lines that did not exhibit SRD (Table 3, Fig. 2g). No significant differences in embryo numbers were observed between the 2 groups. Notably, lines expressing codon-optimized *Smok^Tcr^*, which exhibited the highest distortion effects, also maintained high fertility (Table 3, Fig. 2g). These findings demonstrate that our transgenic males are healthy, fertile, and consistently maintain the SRD effect through generations.

**Table 3.** Fertility parameters of transgenic lines showing sex ratio distortion compared to control lines without effect.

		TgY2	TgY3	TgY4	TgX2	Tg7-2G6 control 1	Tg11-1 control 2	Tg12-1F6 control 3
Males tested		5	4	4	4	5	5^a^	4
Generation		G0, N1	G0	G0	G0	G0	G0	G0
Total plugs		28	22	15	17	Not recorded	18	18
Successful plugs, pregnancies		25	20	13	17	23	13	15
Successful plugs/total plugs		0.89	0.91	0.87	1	*n*.a.	0.76	0.83
Embryos total		275	196	161	232	267	171	158
Embryo per pregnancy, average		11	9.8	12.4	13.6	11.6	13.2	10.5
Embryos per female, std dev between males		1.8	2.4	1.2	1,1	0.9	2.3	2.2
Total embryos for each male (m), average number of embryos per mating, std deviation of embryo numbers per mating	m1	58, 9.7, 5.3	47, 6.7, 3.1	37, 12.3, 1.5	70, 14, 1	51, 12.8, 3.3	22, 11, 1.4	30, 7.5, 2.9
	m2	48, 12, 2.7	46, 11.5, 2.4	39, 13, 1.6	48, 12, 5.8	72, 12, 5	60, 15, 0.7	47, 11.8, 4.8
	m3	57, 14.3, 2.2	33, 11, 2.6	57, 14.2, 1.9	57, 14.3, 1.9	55, 11, 2.9	64, 12.8, 3.5	56, 11.2, 4
	m4	41, 10.3, 4.5	70, 11.7, 2.9	45, 11.3, 4.1	57, 14.3, 2.8	64, 11, 4.8	10, 10, *n*.a.	25, 12.5, 6.4
	m5	71, 11.8, 5.1				25, 12, 2.1	15, 15, *n*.a.	

To test for sex ratio distortion and fertility, each male was mated with a single female for 3 to 5 d per week and plugs were recorded. Females were checked for pregnancy visually and/or by ultrasound examination. Pregnant females were euthanized until d13.5dpc, embryos were dissected and genotyped. Litters for line establishment and maintenance are not included here (note the difference in numbers to Table 2).

^a^Tg11-1: 2 of 5 males (m4, m5) with only one litter.

## Discussion

Here, we present a genetic system for efficient sex ratio distortion in mice, which can be transferred to farm animals. This is the first time that sex ratio distortion in favor of nontransgenic male offspring has been achieved in a mammal by a single transgene. We selected a suitable promoter/enhancer combination, as well as appropriate landing sites for our transgenes, based on histone ChIP-seq experiments in combination with gene expression data. Upon site-specific integration by homologous recombination on the X or Y chromosome, we achieve a sufficiently high level of haploid-specific transgene expression to obtain either predominantly male or female offspring. Corresponding landing sites on the X or Y chromosome of farm animals, as well as suitable regulatory elements, can be identified with the same criteria, allowing the generation of transgenic livestock males.

Achieving sex ratio distortion is most desirable in cattle. Males are preferred for beef production due to their rapid muscle gain. Females of these breeds are used mostly for breeding. Our transgene construct inserted on the X chromosome of transgenic bulls would result in skewing the sex ratio toward nontransgenic males enhancing beef production efficiency and reducing the slaughtering of unwanted calves. Conversely, in dairy farming, inserting our construct on the Y chromosome may boost productivity by increasing the proportion of females, concomitantly reducing the birth of undesired male calves. Similarly, female offspring is preferred in other farm animals, such as the pig, sheep, or goat. A few Y-transgenic males would be sufficient to maintain the herd or flock.

In summary, our method shows a unique combination of advantages and is complementary to alternative approaches (Douglas et al. 2021; Yosef et al. 2025). In particular, when breeding livestock, the absence of the transgene in offspring of the desired sex is of great advantage. In several countries such as Australia, New Zealand, and the United States, such offspring, although derived from a transgenic parent, are termed “null segregant” and regarded as not genetically modified. Adding to the acceptability of our approach, breeder animals and their offspring are scored as healthy. Importantly, transgene activity is maintained over generations and in different genetic backgrounds, underscoring the robustness of our system. The fully preserved fertility and fecundity are particular advantages not present in any of the other approaches published to date. It also suggests that there is scope for further increasing the strength of the distortion effect or tuning it according to particular requirements. Possible strategies include the use of stronger promoters or further optimizations of mRNA stability, translation efficiency, or biochemical activity of the protein.

## Supplementary Material

iyaf246_Supplementary_Data

## Data Availability

Raw and processed sequencing data have been submitted to NCBI GEO under accession number GSE302799. Supplemental material available at GENETICS online.
