# Supplementary material for: Female- or male-biased offspring generated by targeted distortion of sex chromosome transmission in the mouse
Source: Genetics. 2025 Nov 20;233(4):iyaf246. doi: 10.1093/genetics/iyaf246 (PMC13439697; doi:10.1093/genetics/iyaf246)
Supplement: Supplementary file 1 [file iyaf246_supplementary_data.docx]

| **TgY2, TgY3, TgY4** | | | |
| --- | --- | --- | --- |
| *Cypt1* promoter. PCR amplification from genomic DNA | hb1101 | | TCATGGAATACTTTGGTTTCTCCA |
|  | hb1102 | | AGCAGAGCTCTCCAGAGACT |
| *Cypt1* putative enhancer. PCR amplification from genomic DNA | hb1103 | | cctccatgatattgaactcatactca |
|  | hb1104 | | catgtctgggagaaattaaactga |
| *Zfy2* left homology region  Genome coordinates GRCm39/ mm39: Y:2190617-2190840 | | | |
| *Zfy2* right homology region  Genome coordinates GRCm39/ mm39: Y:2190880-2191082 | | | |
| *Zfy2* integration site  Genome coordinates GRCm39/ mm39: Y:2190825-2190895 | | | |
| *Zfy2* guide RNA-left | hb732 | caccGTTGACTCCAATAGTAAACA | |
|  | hb733 | aaacTGTTTACTATTGGAGTCAAC | |
| *Zf*y2 guide RNA-right | hb734 | caccgTGATAACCTTTGCTTACTAT | |
|  | hb735 | aaacATAGTAAGCAAAGGTTATCAc | |
| Full length amplification of transgenes in *Zfy2* integration site. | hb691 | AGCAGGCACACTATCAGGTT | |
|  | hb692 | CCTCCCAAACCCTGTCACAT | |
|  | | | |
| **TgX2** | | | |
| *Akap4* left homology region  Genome coordinates GRCm39/ mm39: X:6958245-6958494 | | | |
| *Akap4* right homology region  Genome coordinates GRCm39/ mm39: X:6958610-6958859 | | | |
| *Akap4* integration site  Genome coordinates GRCm39/ mm39: X:6958491-6958613 | | | |
| *Akap4* guide RNA-left | hb728 | caccGGTATCGATTCTGAAACTAT | |
|  | hb729 | aaacATAGTTTCAGAATCGATACC | |
| *Akap4* guide RNA-right | hb730 | caccgTTCTTTCATTATCCACTGCC | |
|  | hb731 | aaacGGCAGTGGATAATGAAAGAAc | |
| Full length amplification of transgenes in *Akap4* integration site. | hb678 | TGTATTTGTCTCCCTTGGGTCT | |
|  | hb679 | TCACTCTGGGGCTGGAGTTATA | |
|  | | | |
| **Primers for expression analysis by RT-qPCR** | | | |
| TgY2 TgY3, Tcr-t6  122 bp | hb800 | gaatccatctgtgaccccactct | |
|  | hb801 | AGACAGGCACTTCCTTATTGT | |
| TgY4, TgX2  109 bp | hb2150 | CCTGCAAGAAGATGAGGCCA | |
|  | hb2151 | CATGATGTTGTCGGGGGTCA | |
| *Gapdh*  150 bp | hb576 | TGTGTCCGTCGTGGATCTGA | |
|  | hb577 | TTGCTGTTGAAGTCGCAGGAG | |
|  | | | |
| **Genotyping primers** | | | |
| TgY2, TgY3  390 bp transgene band | hb1262 | gccaagctgggtatgcctta | |
|  | hb1261 | TTGACTCCAATAGTAcccgcg | |
| TgY4, tgX2  777 bp transgene band | hb1555 | GTGCACCATCCACCCTAACA | |
|  | hb1969 | TGCATTCTAGTTGTGGTTTGTCC | |
| Sexing primers  X-band 331 bp, Y-band: 302 bp | hb455 | CTGAAGCTTTTGGCTTTGAG | |
|  | hb456 | CCACTGCCAAATTCTTTGG | |
| *t-*haplotype, distal marker: MGI Accession ID: MGI:1327797  t-band: 220 bp, wt-band: 201 bp | hb292-hb1 | GAGTGACCTGCATGCCCACAAGCTGTG | |
|  | hb293-hb2 | GACCTGTGGAGACAGGAAGGGTCAGTG | |
| *t*-haplotype, proximal marker: MGI Accession ID: MGI:3033374  t-band: 195 bp, wt-band: 228 bp | hb459-Vil2-L | TCATGGACCAACACAAGCTC | |
|  | hb460-Vil2-R | CACAAAACTGAAATCTCCCTCTC | |
|  | | | |
| **Probes for in situ hybridization by RNA-scope (ACDBio)** | | | |
| SV40pA-probe Cat#423731 For tgY2, tgY3, tgY4 | | | |
| Tcr-cod.opt probe Cat#1179051-C1 For tgY4 | | | |
| Myc-probe Cat#467601 | | | |
| Polr2a-probe Cat#312471 (positive control) | | | |
|  | | | |
| **Plasmids** | | | |
| pX330-U6-Chimeric_BB-CBh-hSpCas9 | Obtained from Feng Zhang (Addgene plasmid # 42230 ; http://n2t.net/addgene:42230 ; RRID:Addgene_42230) | | |
| pSUPERIOR.puro | novoprolabs | | |

**Supplementary Table 1**

Primers, genomic information, probes and plasmids used in this study.
